# Transcriptome Analysis of Multiple Metabolic Tissues in High-Salt Diet–Fed Mice

**DOI:** 10.3389/fendo.2022.887843

**Published:** 2022-05-17

**Authors:** Fei Mao, E. Wang, Jing Xu, Jin Lu, Guofeng Yan, Li Fu, Yang Jiao, Ling Wu, Tiemin Liu, Yao Li

**Affiliations:** ^1^ School of Life Sciences, Fudan University, Shanghai, China; ^2^ Department of Laboratory Animal Science, School of Medicine, Shanghai Jiao Tong University, Shanghai, China; ^3^ Department of Endocrinology and Metabolism, Zhongshan Hospital, Fudan University, Shanghai, China; ^4^ Department of Assisted Reproduction, Shanghai Ninth People’s Hospital, Shanghai Jiao Tong University School of Medicine, Shanghai, China

**Keywords:** HSD, transcriptome sequencing, metabolism, organokines, lipogenesis

## Abstract

High-salt diet (HSD) is associated with dysregulated metabolism and metabolic disorders. Although previous studies have indicated its effect on metabolic tissues, the involving molecular mechanisms are not quite understood. In the present study, we provided a comprehensive transcriptome analysis on multiple metabolic tissues of HSD-fed mouse model by RNA sequencing. We observed that several genes associated with *de novo* lipogenesis and cholesterol biosynthesis were significantly downregulated in white adipose tissue and liver tissue of HSD mice group, such as *Fasn*, *Scd1*, *Acaca*, and *Thrsp.* Furthermore, combined with secretome datasets, our results further demonstrated that HSD could alter expression levels of organokines in metabolic tissues, for example, *Tsk* and *Manf*, in liver tissue and, thus, possibly mediate cross-talk between different metabolic tissues. Our study provided new insight about molecular signatures of HSD on multiple metabolic tissues.

## Introduction

Salt, a traditionally taste enhancer, has been commonly used in most modern diets and attracted global attention because of its negative effects on human health (1). It has already been well-established that excessive salt intake may increase the risk of hypertension and induce cardiovascular diseases through different mechanisms of organ damage (2). More recently, a high-salt intake has been implicated in the development of chronic inflammation, insulin resistance, and metabolic disorders (3). Dietary sodium overload might affect directly or indirectly on fat distribution and possibly induce liver fibrosis and fatty liver (4–6). However, detailed molecular changes of metabolic organs induced by high-salt diet (HSD) are still unclear.

Adipose, skeletal, and hepatic tissues are the main endocrine organs that produce organokines including adipokines, myokines, and hepatokines, which have been increasingly under study because of close relation to metabolism (7). These organokines can be harmful or beneficial to the organism and perform cross-talk between tissues, acting through endocrine, paracrine, or autocrine pathways (8). For example, the liver transmits information and communicates with other organs including the CNS, adipose tissues, and skeletal muscle, in part, by producing hepatokines (8). In recent years, several hepatokines including Tsukushi (TSK), angiopoietin-like proteins (ANGTPLs), insulin-like growth factors (IGFs), IGF have been identified as information carrier to adipose tissues and skeletal muscles (9). Studies have examined for their roles in lipid and glucose homeostasis. Disruption in the ability of these tissues to communicate manifests as dysregulation of lipid handling and mitochondrial function in the liver, excessive cytokine and lipid release by adipose tissue, and ectopic fat deposition in skeletal muscle, thus resulting obesity and insulin resistance (9). Therefore, understanding the mechanisms involved in the secretory pattern can be useful in the investigation of different diseases. With the advent of the era of omics technology, multi-omics have been increasingly applied to reveal the mechanisms of many human diseases (10).Transcriptomic sequencing technology, also known as RNA sequencing (RNA-seq), has become a powerful tool widely applied to analyze alterations in transcriptional gene expression and enable a new way to unveil specific biological processes (BPs) and underlying novel mechanisms (10).

In our study, we analyzed body metabolism in a high-salt diet mice model by feeding 6-week-old C57BL/6 male mice 12 weeks of 8% NaCl diet. Through whole-transcriptome sequencing on metabolic tissues, including liver, muscle, and white adipose tissue (WAT), we aim to reveal altered patterns of gene expression induced by high-salt diet. We further performed differentially expression analysis and pathway enrichment analysis and screened for all secretomes detected in HSD group compared to control (CON) group. To our knowledge, the comprehensive transcriptome analysis of metabolic tissues of high-salt diet mouse model has not been reported yet. This work demonstrated significantly changed gene expression of metabolic tissues after chronic high-salt intake, indicating possible mechanisms at a molecular level from a new perspective.

## Methods

### Animal Experiments

Six-week-old male C57BL/6 mice were purchased from Lin Chang Laboratory Animal Care, Shanghai, and housed in the Department of Laboratory Animal Science, Shanghai Jiao Tong University School of Medicine, China. One day after arrival, they were habituated for 1 week and then randomly divided into two main groups on normal diet and HSD (8% NaCl), respectively. All mice were housed at 21°C ± 1°C with 55% ± 10% humidity and a 12-h light/12-h dark cycle. Analysis was performed at the end of week 12. After that, mice were anesthetized and sacrificed. Whole liver tissue, epididymal WAT (eWAT), and inguinal WAT (iWAT) were obtained; liver, total eWAT, and total iWAT fat mass weight were recorded. Tissue samples, including liver, WAT, and quadriceps (QU) muscles, were harvested for further analysis.

### Histological Analysis

Formalin-fixed and paraffin-embedded mouse WAT and liver sections were stained. Frozen sections from CON and HSD mice were stained with hematoxylin for 5 min, and Hematoxylin and Eosin (H&E) staining was performed for detection of lipid accumulation in the liver and WAT. Frozen liver sections from HSD and CON mice were stained with 0.5% oil red O staining reagent for 20 min.

### RNA Extraction, Transcriptome Analysis, and qPCR Analysis

RNA was extracted from liver, eWAT, and QU muscle using traditional TRIzol methods. All samples passed through the following three steps before library construction: (i) NanoDrop for RNA purity check, OD260/OD280 in a range of 1.95–2.05; (ii) agarose gel electrophoresis for RNA integrity and potential contamination; and (iii) Agilent 2100 for confirming RNA integrity.

RNA libraries were constructed by using rRNA-depleted RNAs with the TruSeq Stranded Total RNA Library Prep Kit (Illumina, San Diego, CA, USA) according to the manufacturer’s instructions. Libraries were controlled for quality and quantified using the BioAnalyzer 2100 system (Agilent Technologies, Inc., USA). Libraries of 10 pM were denatured as single-stranded DNA molecules, captured on Illumina flow cells, amplified *in situ* as clusters and finally sequenced for 150 cycles on Illumina NovaSeq 6000 platform according to the manufacturer’s instructions. Paired-end reads were harvested from Illumina NovaSeq 6000 sequencer and were quality controlled by Q30. After 3′ adaptor trimming and low-quality reads removing by cutadapt software (v1.9.3), the high-quality clean reads were aligned to the reference genome (UCSC MM10) with hisat2 software (v2.0.4). Then, guided by the Ensembl gtf gene annotation file, cuffdiff software (part of cufflinks) was used to get the gene level FPKM (fragments per kilobase of transcript per million fragments mapped) as the expression profiles of mRNA. Differential expression analysis of two groups was performed using the DESeq2 R package. For RNA-seq, an adjusted false discovery rate (FDR) < 0.05 and |Fold Chang| ≥ 2 were used as the cutoff values for identifying differentially expressed genes (DEGs).

For the quantitative polymerase chain reaction (qPCR) analysis, 1 µg of total RNA was reverse transcribed as cDNA by PrimeScript™RT Master Mix (Takara,RR036A). The qPCR analysis was performed on quantitative realtime PCR system (Roche, LightCycler 480) with SYBR Green Master Mix (Thermo Fisher Scientific, 4309155). Internal control using Gapdh gene for data analysis and cycle threshold (Ct) values was calculated using the 2^ΔΔ-Ct^ method. The primer sequences used in this study are shown in **Supplementary Table 1**.

### Gene Enrichment Analysis

Database for Annotation, Visualization, and Integrated Functional Annotation Tool was used to perform Gene Ontology (GO) terms, including BP, cell component, and molecular function, and Kyoto Encyclopedia of Genes and Genomes (KEGG) pathway enrichment analysis. The top altered GO terms and KEGG pathways are shown in **Supplementary Table 4**. A corrected p-value < 0.05 was considered significantly enrichment.

### PPI Network and Module Analysis

The protein–protein interaction (PPI) network was built by using Metascape for the Retrieval of Interacting Genes. The uploaded genes were clustered into networks to detect significant functional modeules. Cytoscape was used to visualize the PPI network and to calculate the network based on interaction of protein information.

### Statistical Analysis

Data are expressed as the mean ± SEM, and statistical analyses were completed using GraphPad 8.0. Student’s t-test was used to compare the difference between two groups, and differences were considered statistically significant at p < 0.05.

## Results

### Long-Term High-Salt Intake Decreases Fat Deposition in Adipose Tissue of C57BL6 Mouse

**Figure 1:**
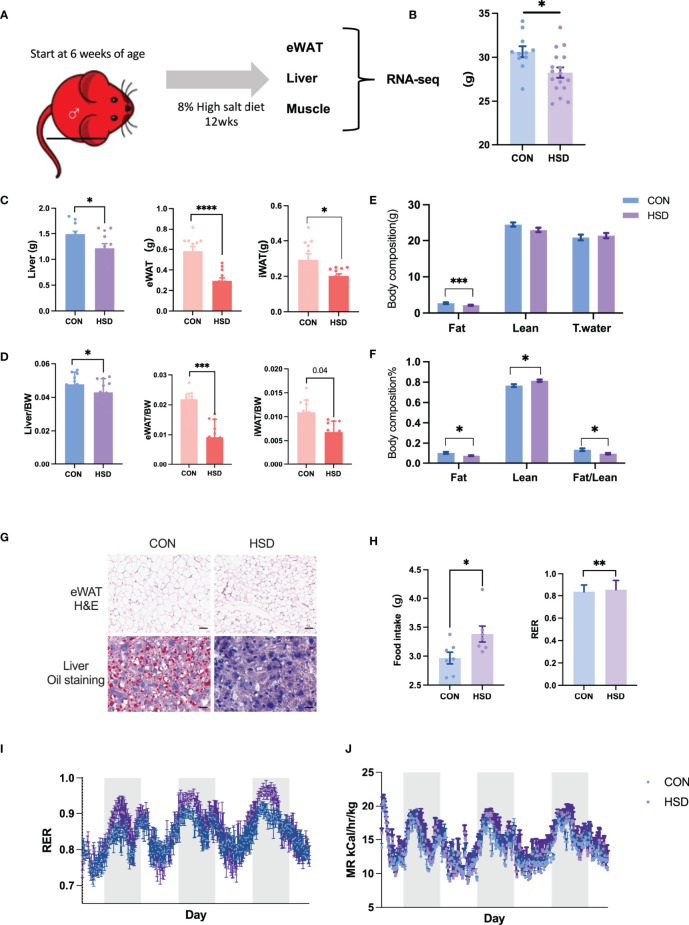
Metabolic parameters of 12 weeks high-salt diet–induced mouse model. **(A)** Workflow of RNA-seq in three metabolic tissues of high-salt diet–induced mouse model. **(B)** Body weight, **(C)** metabolic tissue weight, **(D)** tissue to body weight, and **(E, F)** body composition measured in HSD and CON mice. **(G)** Representative H&E and oil red O staining of liver and eWAT of HSD mice group compared to CON group. **(H)** Feed intake and RER level of HSD and CON mice group. **(I, J)** RER and metabolic rate detected by metabolic cage of HSD and CON mice group. Data are presented as mean ± SEM. ^*^ *P* < 0.05, ^**^ *P* < 0.01, ^***^ *P* < 0.001, and ^****^ *P* < 0.0001 compared to the control group. The scale bar represents 50 µm. n = 6–10 per group.

It has been reported that high-salt diet could alter fat deposition and liver metabolism accompanied by metabolic changes. To understand the detailed changes of metabolic tissues induced by high-salt diet, we established mice models by feeding mice with 8% high-salt diet and 0.9% saline for 12 weeks (8% NaCl + 0.9% saline water, as the HSD group). Their control group of mice was fed with chow diet and tap water for 12 weeks (CON) (**Figure 1A**). After 12 weeks of high-salt diet, urine Na^+^ output and aldosterone level of mice were significantly altered as reported by previous study (**Supplementary Figure 1**). Interestingly, we found body weight of HSD group of mice decreased by 6% compared with CON group of mice (**Figure 1B**). Liver weight and fat mass of HSD mice decreased compared with CON mice by 20% and 40%, respectively (**Figures 1C, D**). Of note, eWAT mass decreased most significantly in HSD mice compared to CON by 50%. Body composition analysis further confirmed the result (**Figures 1E, F**). H&E staining of WAT tissue revealed decreased adipocyte volume, and liver oil staining revealed decreased lipid accumulation in HSD group of mice (**Figure 1G**). These results indicated that HSD intake could negatively regulate fat deposition of male mice. Feed intake and body metabolism were measured for 3d using CLAMS after HSD and CON diets were fed for 12 weeks. Feed intake and respiratory exchange ratio (RER) of HSD group were significantly higher than CON group (**Figures 1H, I**). However, metabolic rate in the HSD mice remained unchanged (**Figure 1J**). To comprehensively understand the molecular signature of metabolic tissues in HSD-fed mice, compared to their age matched controls, we further performed RNA-seq of liver, WAT, and QU muscle samples.

### Long-Term High-Salt Intake Changes RNA Expression in Different Metabolic Tissues

Because eWAT mass showed more decrease in mass than iWAT mass in HSD group with no significant change in energy expenditure, we chose to focus on eWAT for further investigation. After RNA was extracted from liver, muscle, and eWAT tissue, transcriptome sequencing was performed to analyze the transcriptional changes of gene expression. The RNA-seq data for all samples have been deposited in the GEO database with accession number GSE197972. The quality of mRNA-seq data from all samples is summarized in **Supplementary Table 2**, with Q30 for all samples above 90% of reads. Normalized gene counts were detected after batch-effect correction by interquartile range boxplot, and Principal Component Analysis (PCA) analysis shows normalized read counts in each mouse sample. Gene expression pattern was demonstrated by the hierarchical clustering analysis and heatmap, in which, samples among each group are closely clustered, which indicated a higher repeatability in biological replication (**Supplementary Figure 2**). During the identification of DEGs, a fold change ≥ 2 and FDR < 0.05 were used as screening criteria. Through differential analyses, we identified altogether 653 DEGs in the liver tissue of high-salt group, of which 354 were upregulated and 299 were downregulated. We identified DEGs in the eWAT tissue of high-salt group, of which 667 were upregulated and 329 were downregulated (**Table 1**). In addition, we identified altogether 57 DEGs in muscle tissues, most of which were downregulated. In the liver tissue, DEGs included the genes encoding for lipid metabolism (such as *Fasn*, *Acaca*, *Acyl*, and *Thrsp*) were found downregulated (**Table 2**). DEGs in eWAT tissue including several key lipogenic genes (such as *Fasn*, *Scd1*, *Acaca*, and *Thrsp*) and fatty acid transport genes (such as *Fabp1* and *Fabp5*) were also found downregulated. All of DEGs listed in enriched pathways were listed in **Supplementary Table 3**. To confirm the changes in gene expression determined by RNA-seq, we performed quantitative real-time quantitative PCR (RT-qPCR) assays for representative genes in lipogenic and fatty acid metabolism pathways from HSD group to validate mRNA-seq data (**Supplementary Figure 2**).

**Table 1:** DEGs detected in different metabolic tissues from RNA-seq in HSD group.

CON	HSD	Upregulated	Downregulated	Total DEGs
Liver	Liver	354	299	653
eWAT	eWAT	667	329	996
Muscle	Muscle	14	43	57

DEGs, differentially expressed genes; CON, Control; HSD, high-salt diet.

**Table 2:** Key DEGs significantly changed in metabolic tissues of HSD group.

eWAT		Liver		Muscle	
HSD vs. CON		HSD vs. CON		HSD vs. CON	
*Gene Name*	Log2 Fold Change	*Gene Name*	Log2 Fold Change	*Gene Name*	Log2 Fold Change
*Fasn*	−3.60	*Thrsp*	−4.31	*Fbxo32*	1.45
*Acaca*	−2.11	*Dhcr7*	−1.91	*Myom3*	−1.01
*Scd1*	−1.52	*Lpin1*	−2.09		
F*abp5*	−1.47	*Acaca*	−1.31		
*Fabp1*	−4.56	*Fasn*	−2.53		
*Thrsp*	−2.13	*Mvd*	−1.62		

DEGs, differentially expressed genes; CON, control; HSD, high-salt diet.

### Altered Level of Hepatokines and Reduction of Lipid Metabolism in Liver of HSD-Fed Mice

**Figure 2:**
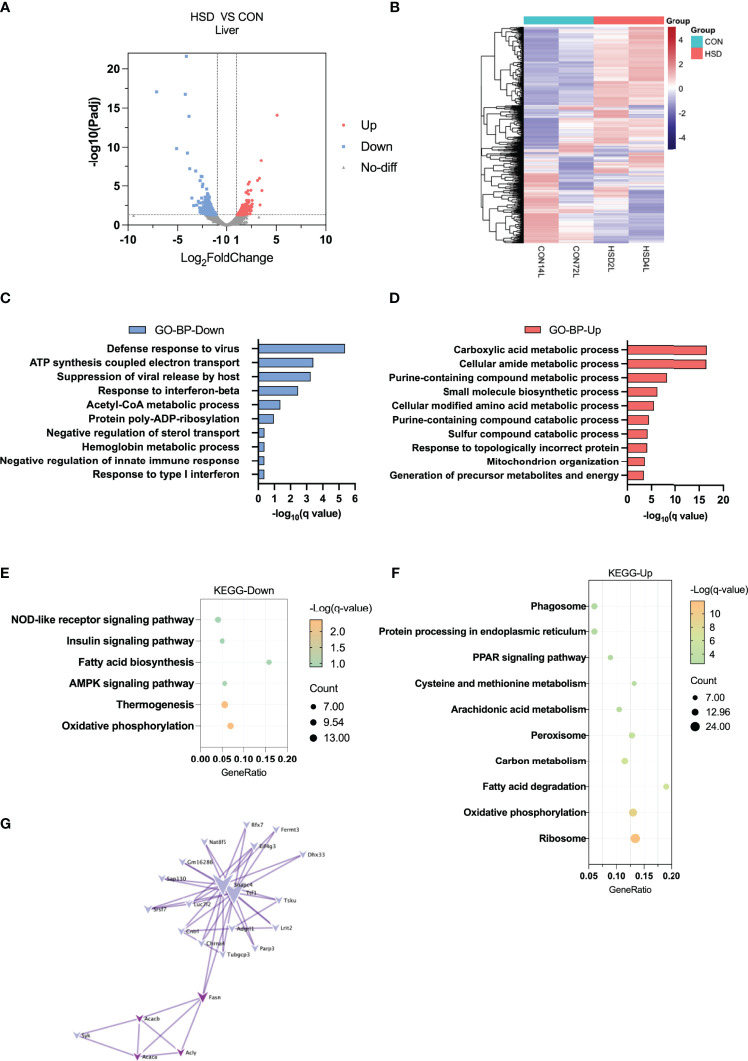
Transcriptome analysis of liver tissue in the high-salt diet (HSD) vs. CON groups of mice. **(A)** Volcano plot showing DEGs (up- and downregulated) from liver tissue in HSD group compared with CON group. The red dots represent the upregulated genes, and the blue dots represent the downregulated genes. **(B)** Heatmap showing gene expression patterns in the liver of HSD group compared with CON group. **(C, D)** Gene Ontology analysis of the biological process of upregulated **(C)** and downregulated **(D)** differential genes from liver tissue in HSD group compared to CON group. **(E, F)** KEGG analysis assessing the pathways associated with the upregulated **(E)** and downregulated **(F)** gene sets from liver tissue in HSD group compared to the CON group. **(G)** Protein–protein interaction analysis of downregulated differential genes in the liver of HSD group mice.

Heatmap and volcano plot showed most DEGs between HSD and CON group of liver tissue (**Figures 2A, B**). To explore the possible functions, these DEGs, GO, and KEGG enrichment analysis were conducted among these DEGs induced by HSD. The GO enrichment analysis of BP suggested that defense response to virus, response to interferon-beta, acetyl-CoA metabolic process, and negative regulation of sterol transport were downregulated; whereas carboxylic acid metabolic process and cellular amide metabolic process were upregulated in the liver of HSD group (**Figures 2C, D** and **Supplementary Table 4**). Consistently, the KEGG pathway analysis revealed that the AMPK signaling pathway and fatty acid biosynthesis were downregulated in liver of HSD group; whereas fatty acid degradation was upregulated (**Figures 2E, F** and **Supplementary Table 4**). Furthermore, we analyzed PPIs for downregulated genes with a PPI network and found that lipid synthetic genes like *Fasn*, *Acc1*, and *Acly* showed decreases in HSD group of mice (**Figure 2G**).

The liver has recently been recognized as an endocrine organ that secretes hepatokines, which are liver-derived factors that can signal to and communicate with distant tissues. We discovered in our study that expression level of several reported hepatokines was altered in the liver of HSD mice, including *Tsku*, *Manf*, and *Igfbp2* (**Supplementary Table 5**). Previous studies have already indicated that *Tsku* could likely improve energy homeostasis by regulating thermogenesis, level of which was induced in insulin resistance. In addition, *Manf* is also a newly reported feeding induced hepatokine, which could ameliorate diet-induced obesity by promoting adipose browning. Thus, our data suggested that HSD is probably associated with altered level of hepatokines and lipid metabolism in liver tissue.

### Decreased Lipid Metabolism and Reduced Lipogenesis in eWAT of HSD Group of Mice

**Figure 3:**
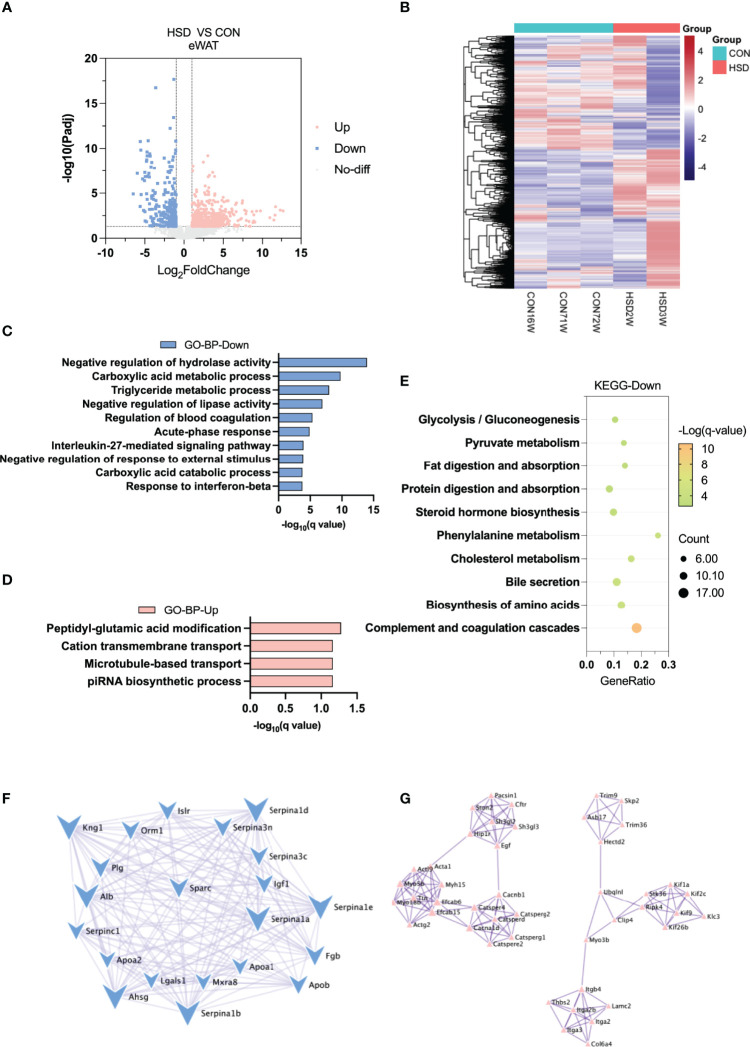
Transcriptome analysis of eWAT in the high-salt diet (HSD) vs. CON groups of mice. **(A)** Volcano plot showing DEGs (up- and downregulated) from eWAT in HSD group compared with CON group. The red dots represent the upregulated genes, and the blue dots represent the downregulated genes. **(B)** Heatmap showing gene expression patterns in eWAT of HSD group compared with CON group. **(C, D)** Gene Ontology analysis of the biological process of upregulated **(C)** and downregulated **(D)** differential genes from eWAT in HSD group compared to CON group. **(E)** KEGG analysis assessing the pathways associated with downregulated gene sets from eWAT in HSD group compared to the CON group. **(F, G)** Protein–protein interaction analysis of upregulated **(F)** and downregulated **(G)** differential genes in eWAT of HSD group mice.

Heatmap and volcano plot showed most DEGs between HSD and CON group of liver tissue (**Figures 3A, B**). In eWAT of HSD mice, GO analysis of BP showed that negative regulation of hydrolase activity, carboxylic acid metabolic process, and triglyceride metabolic process were downregulated (**Figures 3C, D**). KEGG pathway analysis also confirmed that HSD could downregulate cholesterol metabolism and increase fatty acid degradation (**Figure 3E** and **Supplementary Table 4**). PPI network analysis of DEGs further showed that genes involved in seretomes including *Apoa2*, *Apoab*, *Serpins*, and *Alb* were mainly downregulated in eWAT of HSD induced mice group; whereas upregulated genes were distributed in different scattered clusters (**Figures 3F, G**). However, GO and KEGG analysis showed no significant pathways enriched in muscle tissue of HSD, indicating the effect of HSD on muscle tissue was minimal (**Supplementary Figure 3**). Our results suggested a decrease in lipid metabolism and a reduction of lipogenesis in eWAT of HSD mice group.

### Identification of Secretomes Distinctively Altered in Metabolic Tissues of HSD Groups of Mice

**Figure 4:**
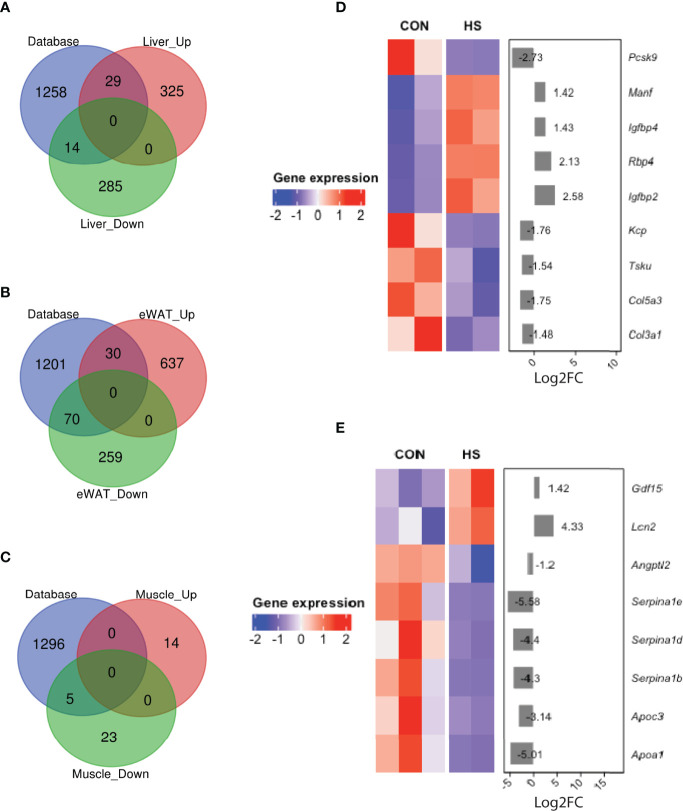
DEGs of secretomes detected in HSD mouse metabolic tissues. **(A–C)** Venn diagram showing organokines detected by overlapping DEGs with mouse secretome database in **(A)** liver, **(B)** eWAT, and **(C)** muscle tissue of HSD mouse model. **(D, E)** Key secretomes differentially expressed in liver tissue **(D)** and eWAT **(E)** of HSD mice group.

To identify possible role of organokines involved and further understand the possible cross-talk between metabolic organs induced by HSD, we overlapped DEGs in three metabolic tissues with a mouse secretome database (NCBI GEO, accession number: GSE54650) (**Figure 4** and **Supplementary Table 5**). Notably, 29 upregulated and 14 downregulated hepatokines were detected in liver tissue from HSD group of mice compared to their controls (**Figure 4A**). Among these genes, *Tsk*, *Manf*, and *Kcp* have been reported to be involved in energy expenditure and metabolic disorders (**Figure 4D**). Together, a total of 30 adipokines were detected upregulated and 70 downregulated in eWAT of HSD group (**Figure 4B**). Among them, *Lcn2* and *Angptl2* have been reported to promote inflammation and insulin resistance (**Figure 4E**). Meanwhile, a total of seven myokines were detected significantly downregulated in the muscle of HSD group (**Figure 4C**); however, none of them was reported in muscle BP.

## Discussion

It is already well known that high-salt intake is harmful to human health. Recent studies showed that a high intake of dietary salt (NaCl) has been implicated in the development of hypertension, chronic inflammation, and autoimmune diseases (11, 12). Previous studies have already testified that high-salt diet could activate certain immune cells and thus exacerbate experimental models of autoimmunity (11, 13, 14). As is known to all, metabolic disorders, such as obesity, non-alcoholic steatohepatitis (NASH), and others, are closely related to chronic inflammation (15). For example, inflammation is one of the hallmarks of NASH, the progressive state of non-alcoholic fatty liver disease (NAFLD) (15). The endocrine function of the liver, adipose, and hepatic tissues is of great value in NASH development. These tissues can produce different biomarker peptides, namely, organokines (including hepatokines, adipokines, and myokines, respectively) that cross-talk through autocrine, endocrine, and paracrine pathways (8, 16). The combined action of organokines is related to health or to the pathological process of several diseases (8). With the increase in adipose tissue mass, there is an increase in the secretion of pro-inflammatory organokines, contributing to metabolic disorders such as insulin resistance and metabolic syndrome (9). Two major goals of this study were i) to gain better understanding of the effect of high-salt intake on metabolism and ii) to provide clues of a possible regulatory mechanism underlying HSD induced metabolic changes.

In our study, we used a mice model fed with 8% high-salt diet plus 0.9% saline, examined metabolism at the whole-body level, and used transcriptome analysis to expose mechanisms at the molecular level. As we expected, HSD could inhibit RAAS (renin angiotensin aldosterone system) level and significantly increase urine Na^+^ output. Interestingly, we found that fat deposition in liver and WAT both decreased following *ad lib* feeding of the HSD diet for 12 weeks. According to previous clinical and preclinical studies, HSD has certain role in the development of NAFLD (4, 6, 17). We assumed that the discrepancies between our findings with previous studies might due to different high-salt diet composition. On the basis of calculation of daily food and water intake, the total daily sodium intake was increased by 20-fold in our HSD group compared to normal diet group. Because daily sodium intake was much higher in our study than in previous study, it might result in different metabolic status as well. However, HSD group of mice showed no significant difference in energy expenditure even with more feed intake and decreased body weight, suggesting that salt intake alone might have a lesser impact on body metabolism compared to other diets such as high fat or western diet.

In our RNA-seq results of liver, we found genes related to lipogenesis were significantly decreased in mice of HSD group, including those involved in fatty acid and lipid biosynthesis and transport (*Fasn*, *Scd1*, *Acaca*, *Fabp1*, *Fabp5*, etc.). We further identified the expression changes of several reported hepatokines changed by HSD. Of note, the level of a well-known hepatokine, Tsukushi (TSK), decreased by 2.8-fold change after 12 weeks of HSD. TSK is an inducible hepatokine that regulates energy expenditure least, in part, through brown fat sympathetic innervation (18). Hepatic and plasma levels of TSK were strongly induced by different stimuli that increased thermogenesis and energy expenditure, including adrenergic agonists and cold exposure (19). Apart from TSK, another hepatokine, *Manf*, also attracted our attention due to its role in energy expenditure. Mesencephalic astrocyte–derived neurotrophic factor (*Manf*) is a feeding-induced hepatokine, which plays a crucial role in regulating thermogenesis in adipose tissue (20). However, the expression levels of TSK and *Manf* were inconsistent with each other in the liver of HSD mice, suggesting a more complex cross-talk by secretomes between metabolic tissues in HSD mice model.

Another finding in our study is a decrease of WAT volume induced by HSD. Previous study has indicated that HSD diet could depress lipogenesis, thereby contributing to reduced fat deposition in mouse adipose tissue (5). Although related study was limited, our result again verified this phenotype. Further GO and KEGG analysis testified that lipid metabolism pathways were influenced by high-salt diet in both eWAT and liver tissues. Overlapped with secretome database, we discovered altered level of adipokines in eWAT of HSD mice group, including *Lcn2* and *Angptl2*. Both lipocalin 2 and Angptl were found at high levels in the adipose tissues of diet-induced or genetically obese mice, as well as those of obese individuals (21). In addition, we identified a significant decrease of Serpin genes (including *Serpina3b*, *Serpina3c*, *Serpina1b*, *Serpina3k*, *Serpina1d*, and *Serpina1e*) in eWAT of HSD mouse. Serpins are a superfamily of proteins characterized by their function as serine protease inhibitors (22). So far, a total number of 36 serpins have been identified (23, 24). These proteins are expressed in all the organs and are involved in multiple important functions such as hormone transportation, mediating insulin sensitivity, and inflammatory response (22, 23). Previous studies indicated the association of deficiency or overexpression of certain types of serpins with diverse pathophysiological events (24). Of note, Serpina3c, which has been reported as a critical factor regulating adipogenesis by modulating IGF1 and intergrin signaling pathway (25), was detected down regulated in the eWAT of HSD mouse. We found very few DEGs in the muscle tissue of HSD mice group. Further pathway analysis failed to enrich pathway, indicating HSD has little impact on muscle tissue.

Although we failed to map DEGs to disease ontology pathway due to our special diet model (data not shown), the results of RNA-seq in our high-salt mouse model are in well accordance with phenotypes observed in our study. Our comprehensive transcriptional sequencing analysis provided a new sight into HSD on daily health and metabolism and the underlying molecular mechanisms it correlates. However, several limitations should be pointed out in our study. First, the HSD mice model used in our study could not mimic high-salt diet in our daily life. Second, our present study is a descriptive study; functional experiments should be carried out to testify the underlying mechanisms. However, as far as we know, our study is the first to provide clues of high-salt diet–induced metabolic effects from transcriptome levels and thus might provide theoretical evidence and also some new insights for understanding high-salt diet and its related effects apart from adverse cardiovascular outcomes.

## Conclusions

Our study investigated the mechanisms of changed gene expression pattern of metabolic organs induced by high salt in terms of whole transcriptome profiling.

## Data Availability Statement

The data presented in the study are deposited in the GEO repository, accession number GSE197972.

## Ethics Statement

The animal study was reviewed and approved by Shanghai Jiaotong University.

## Author Contributions

YL, TL, and LW participated in the conception and design of the study. FM and JX carried out animal handling in the study. JL, GY, and LF provided help in animal handling. FM and EW carried out data analysis and interpretation of data and wrote the manuscript. EW and YJ provided help in data interpretation. TL and YL revised the manuscript. All authors contributed to the article and approved the submitted version.

## Funding

This work is supported by National Key Research and Development Program of China (2018YFA0800402), National Natural Science Foundation of China (82100847 and 81900727), and Fund for Excellent Young Scholars of Shanghai Ninth People’s Hospital, Shanghai JiaoTong University school of Medicine (No. JYYQ004).

## Conflict of Interest

The authors declare that the research was conducted in the absence of any commercial or financial relationships that could be construed as a potential conflict of interest.

## Publisher’s Note

All claims expressed in this article are solely those of the authors and do not necessarily represent those of their affiliated organizations, or those of the publisher, the editors and the reviewers. Any product that may be evaluated in this article, or claim that may be made by its manufacturer, is not guaranteed or endorsed by the publisher.
