# Supplementary figures and images for: Transcriptome Analysis of Multiple Metabolic Tissues in High-Salt Diet–Fed Mice
Source: Front Endocrinol (Lausanne). 2022 May 17;13:887843. doi: 10.3389/fendo.2022.887843 (PMC9152432; doi:10.3389/fendo.2022.887843)

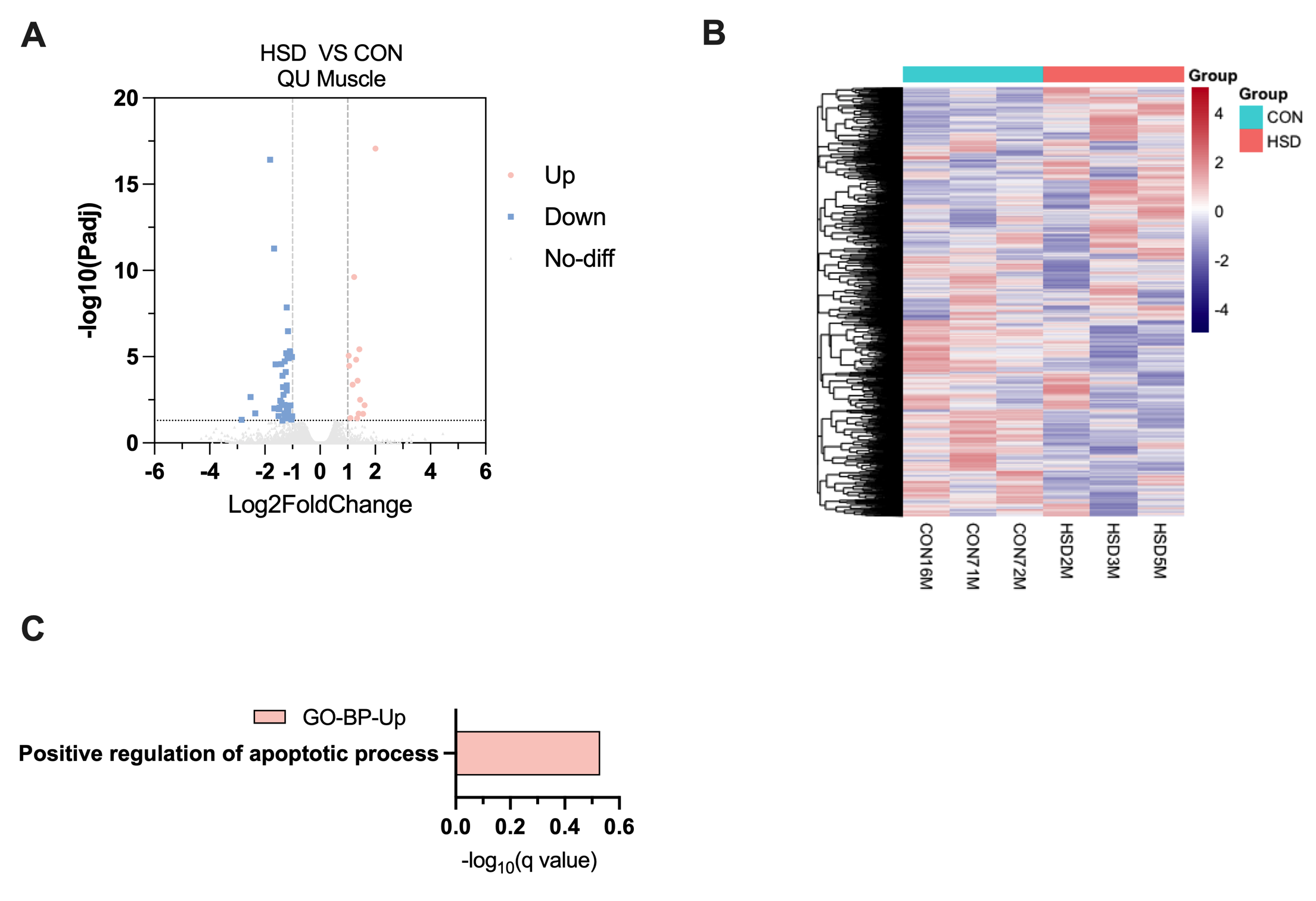

Supplement: Supplementary file 1 [file Image_1.tiff]

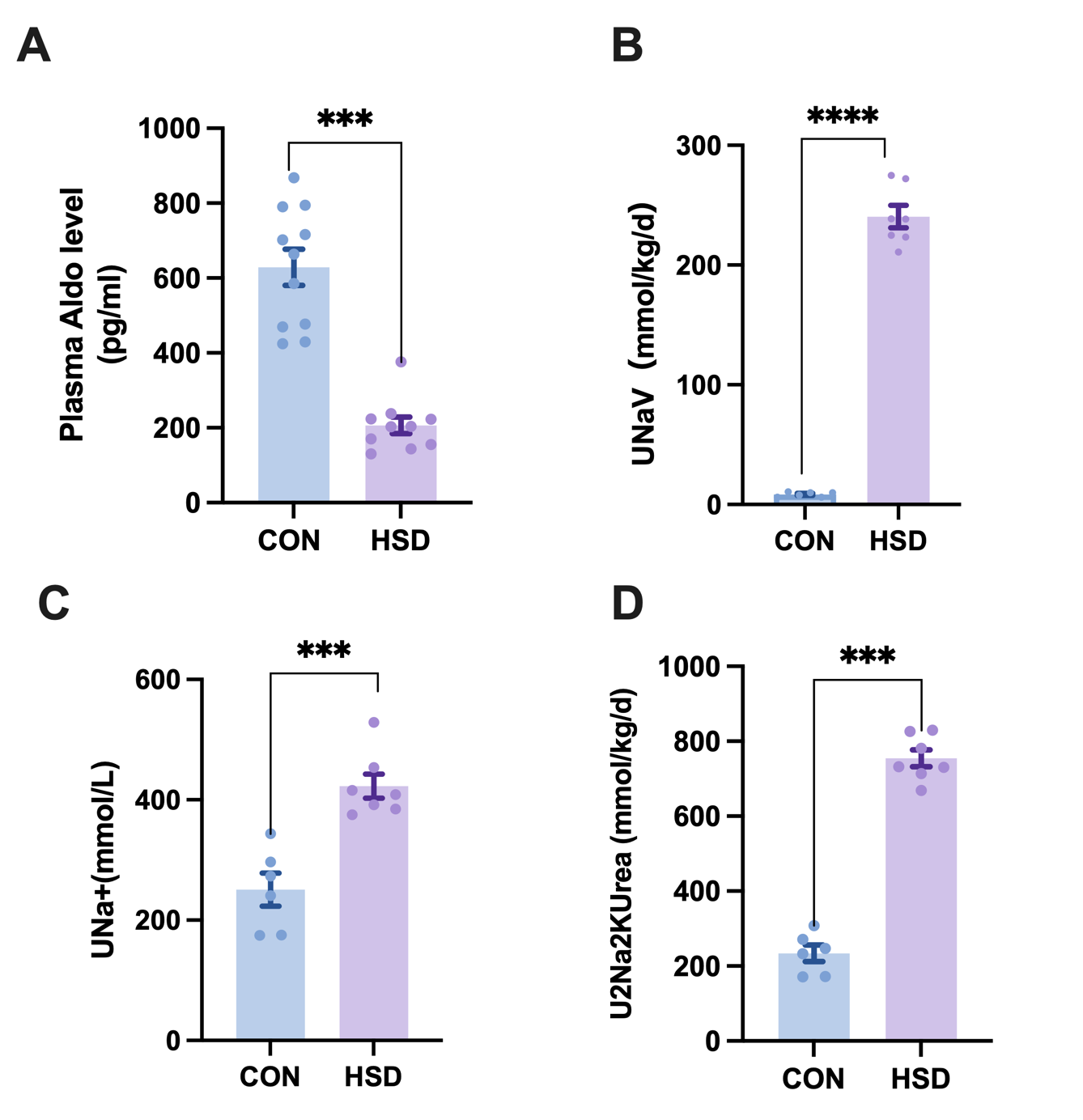

Supplement: Supplementary file 2 [file Image_2.tiff]

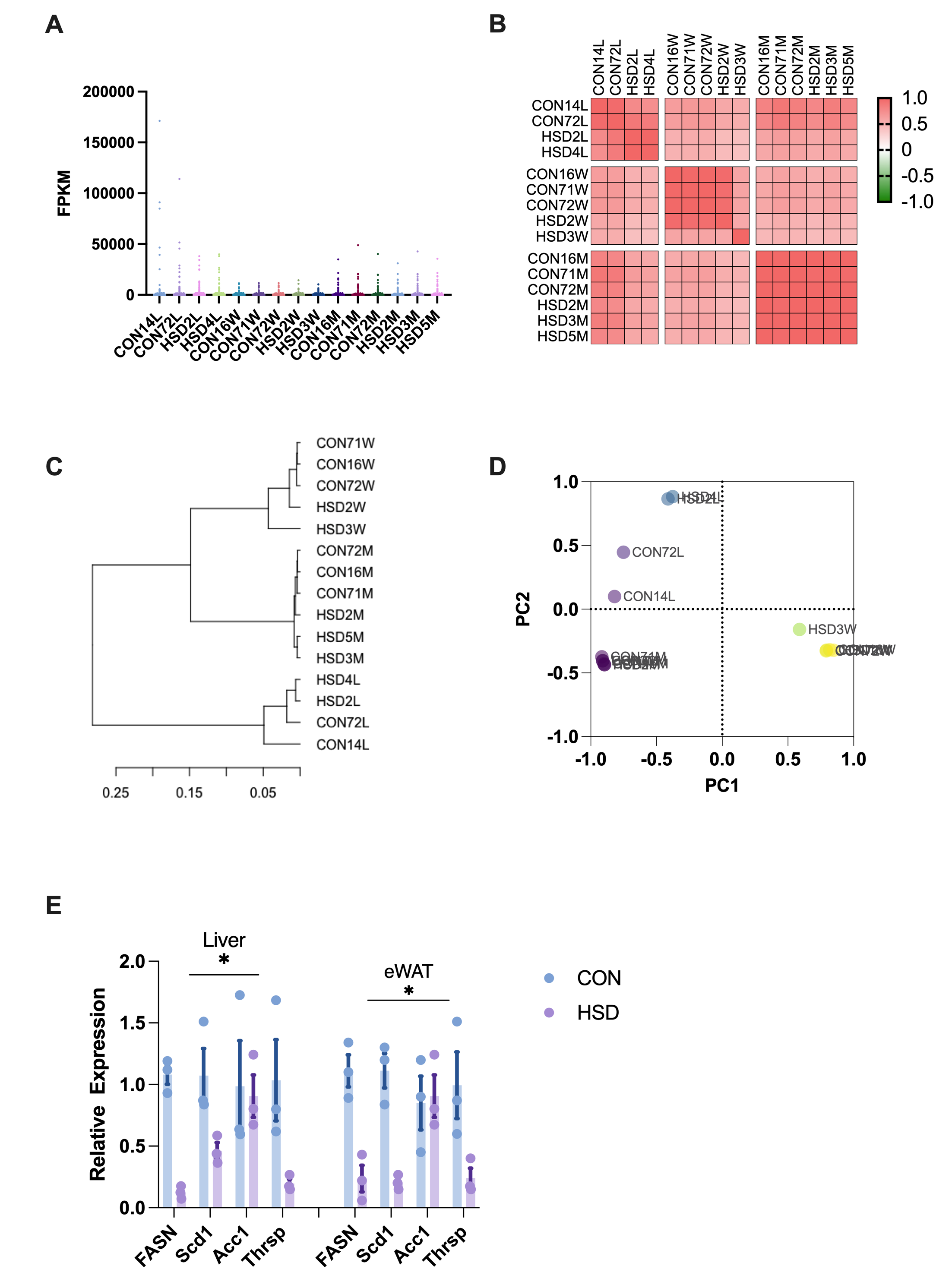

Supplement: Supplementary file 3 [file Image_3.tiff]
